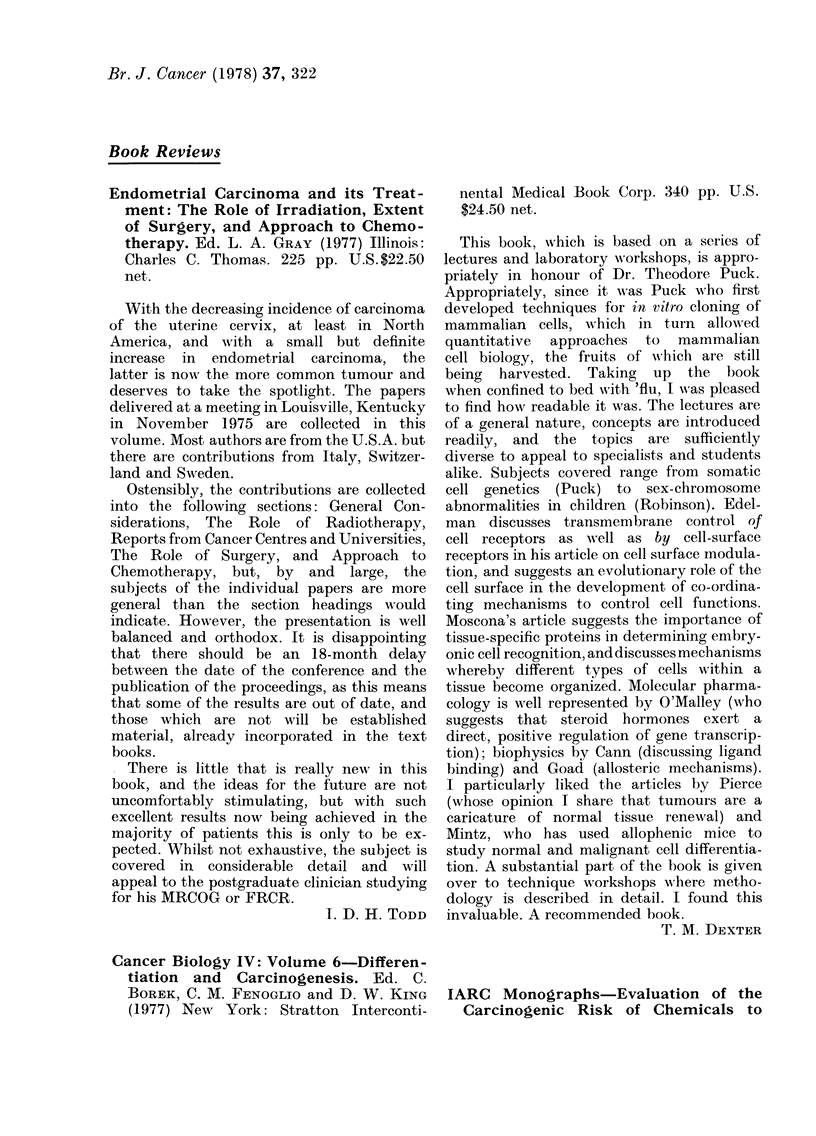# Endometrial Carcinoma and its Treatment: The Role of Irradiation, Extent of Surgery, and Approach to Chemotherapy

**Published:** 1978-02

**Authors:** I. D. H. Todd


*Br. J. Cancer* (1978) **37**, 322

### *Book Reviews*

**Endometrial Carcinoma and its Treatment: The Role of Irradiation, Extent of Surgery, and Approach to Chemotherapy.** Ed. L. A. GRAY (1977) Illinois: Charles C. Thomas. 225 pp. U.S.$22.50 net.

With the decreasing incidence of carcinoma of the uterine cervix, at least in North America, and with a small but definite increase in endometrial carcinoma, the latter is now the more common tumour and deserves to take the spotlight. The papers delivered at a meeting in Louisville, Kentucky in November 1975 are collected in this volume. Most authors are from the U.S.A. but there are contributions from Italy, Switzerland and Sweden.

Ostensibly, the contributions are collected into the following sections: General Considerations, The Role of Radiotherapy, Reports from Cancer Centres and Universities, The Role of Surgery, and Approach to Chemotherapy, but, by and large, the subjects of the individual papers are more general than the section headings would indicate. However, the presentation is well balanced and orthodox. It is disappointing that there should be an 18-month delay between the date of the conference and the publication of the proceedings, as this means that some of the results are out of date, and those which are not will be established material, already incorporated in the text books.

There is little that is really new in this book, and the ideas for the future are not uncomfortably stimulating, but with such excellent results now being achieved in the majority of patients this is only to be expected. Whilst not exhaustive, the subject is covered in considerable detail and will appeal to the postgraduate clinician studying for his MRCOG or FRCR.

I. D. H. TODD